# Associations between health-related self-efficacy and suicidality

**DOI:** 10.1186/s12888-018-1705-z

**Published:** 2018-05-10

**Authors:** Vivian Isaac, Chia-Yi Wu, Craig S. McLachlan, Ming-Been Lee

**Affiliations:** 1 Flinders Rural Health South Australia, Flinders University, Renmark, Australia; 2 School of Nursing, National Taiwan University College of Medicine, Taipei, Taiwan; 3 Taiwan Suicide Prevention Center, Taipei, Taiwan; 4 Rural Clinical School, University of New South Wales, Sydney, Australia; 5 Departments of Psychiatry, National Taiwan University College of Medicine & National Taiwan University Hospital, Taipei, Taiwan; 6 Department of Psychiatry, Shin Kong Wu Ho-Su Memorial Hospital, Taipei, Taiwan

**Keywords:** Self-efficacy, Suicidality, Computer-assisted telephone interview, Taiwan

## Abstract

**Background:**

Few studies have focused on exploring the association of self-efficacy and suicidal behaviour. In this study, we aim to investigate the association between health-related self-efficacy and suicidality outcomes, including lifetime/recent suicidal ideation, suicidal attempts and future intent of suicide.

**Methods:**

A computer-assisted telephone interview (CATI) system was used to draw potential respondents aged over 15 in Taiwan via telephone numbers, which were selected by a stratified proportional randomization method according to the distribution of population size in different geographic areas of Taiwan. We obtained available information on suicide behaviours for the analysis of 2110 participants. Logistic regression was applied to investigate the independent effect of health-related self-efficacy on life-time suicidal thoughts and attempts.

**Results:**

Suicidality measured as suicide ideation and attempted suicide was reported as 12.6 and 2.7% respectively in the sample. Among those with suicide ideation, 9.8% had thoughts of future suicide intent. Female gender, low education, people living alone or separated, history of psychiatric disorders, substance abuse, poor self-rated mental health and physical health were associated with suicidality factors. Low health-related self-efficacy was associated with lifetime suicide ideation, prior suicide attempt and future suicidal intent. Among those with recent suicidal ideation, low health self-efficacy was independently associated with future suicide intent after adjustment of gender, age, education, marital status, substance abuse, psychological distress, poor mental and physical health.

**Conclusion:**

Health-related self-efficacy was associated with suicide risks across different time points from prior ideation to future intention. Evaluation of the progress of self-efficacy in health may be long-term targets of intervention in suicide prevention strategies.

## Background

The suicide rate in Taiwan has been decreasing since 2010 from a high rate of 16.8 to 15.7 per 100,000 in 2015 according to national statistical data. Suicide poses a significant social and economic burden [1]. Developing strategies that target suicide behaviours may improve suicide rates. Suicide behaviour carries a 10–15% lifetime risk of death [2, 3]. Psychiatric morbidities such as depression and anxiety are strongly associated with suicidal ideation and suicide death in Taiwan [2, 4]. Prior suicide attempts are known to be associated with a higher level of engagement of mental health services [5]. The possible risk factors leading to suicide ideation include substance use [6], gender, aging, divorce and unemployment [7].

Suicidality is a complex multifactorial process. It is not known whether self-efficacy impacts on suicide behaviours in the cultural context of Taiwan. Health confidence and health-related self-efficacy are adaptive cognitive factors that have been shown to improve health behaviours [8]. A higher sense of self-efficacy and control has consistently shown to predict better positive health outcomes [9, 10]. Numerous studies have adopted health practices that have measured self-efficacy with respect to behavioural changes. For example, self-efficacy has been measured in chronic disease management, diet, exercise, and tobacco control [11–13].

Only a few studies have globally focused on the association of self-efficacy and suicidal behaviour [14]. A community-based household survey in rural Japan showed lower general self-efficacy was associated with increased rates of suicidal ideation. Feng et al. has proposed that general self-efficacy functions as a mechanism of self-confidence to cope with challenging life stresses [15]. In this study, we aim to investigate the independent association between health-related self-efficacy and suicidal trajectories/outcomes including lifetime suicidal ideation, suicidal attempts and future intent of suicide. These aims were explored in a representative sample throughout the island of Taiwan.

## Methods

### Participants and procedure

Population study sampling was performed via the project administrator of Taiwan Suicide Prevention Centre (TSPC). Specifically, participants were contacted upon sampling via a telephone survey on population mental health, knowledge and behaviour of suicide prevention carried out by the Centre. The ethical approval was acquired from the general hospital the corresponding author affiliates (reference number 201204034RIC). The survey recruited a representative random sample of the general population aged over 15 in Taiwan. Data were collected between July 14th and 23th in 2015 by personnel with specific training for this survey. A computer-assisted telephone interview (CATI) system was used to draw potential respondents via telephone numbers selected by a stratified proportional randomization method according to the distribution of population size, gender and age in different geographic areas of Taiwan. In total, initially, 21,384 subjects landline phone numbers were randomly selected, and 5430 respondents aged 15 years or older were contacted, with 2110 respondents agreed to take part in the survey anonymously over the phone and accomplished the interview (with sampling error of ± 2.10% in 95% confidence interval).

### Measurements

The questionnaire used in the interview included demographics (age, gender, education level, occupation and marital status) and health-related bio-behavioural measures (self-rated physical/mental health, self-efficacy), and suicide risk factors (suicide-related items, psychopathology, and substance use). Definition and assessment for these key variables are listed below.

Suicide ideation/intention to suicide: We evaluated whether the respondents had had previous suicide ideation across different time points; lifetime and in the past week. We also assessed whether they may have future suicide intent through the question, “Is it likely that you may attempt suicide in the future?”

Suicide attempts: The respondents were asked whether they had engaged in a suicide attempt and when this occurred. We determined if this attempt ever happened in their lifetime (classified as a previous attempt).

Health-related measures: A single item question was used to assess health-related self-efficacy based on standard methodology for measuring self-efficacy beliefs [16]. The participants were asked, “How much confidence, from a scale of 0 to 100, do you think you have to control over your own health conditions?” We also inquired about self-rated health conditions in both physical and mental aspects, with the ratings from 0 (very poor) to 4 (very good) [17].

Psychopathology assessment: We used the Brief Symptom Rating Scale to measure the level of psychological distress in the past week of the respondents [18]. It is a 5-item Likert scale (scores of 0 to 4) that contained the following questions [2]: (1) having trouble falling asleep (insomnia); (2) feeling tense or keyed up (anxiety); (3) feeling easily annoyed or irritated (hostility); (4) feeling low in mood (depression); and (5) feeling inferior to others (inferiority).

Substance abuse: A single question was used to assess whether a previous substance misuse leading to life impairments had occurred due to any illicit drug, prescription medication, or alcohol. Substance abuse during any period of lifetime was recorded as a binary response of yes or no based on self-report.

### Data analysis

Descriptive statistics of demographic variables are presented. The following tests were used for data analyses. Univariate and bivariate tabulation was conducted as a prerequisite for multivariate analyses. Associations between independent variables and lifetime suicidal ideation, suicide attempt and future suicide intent were performed using chi-square tests and then were presented as odds ratios (ORs) with 95% confidence intervals (CIs). Self-efficacy score was significantly skewed, therefore the score was log-transformed for further analysis. In order to identify low health-related self-efficacy, a tertile spit of the self-efficacy scale was conducted. The participants in the lower tertile (represented a score of below 80) were categorised as having low health self-efficacy. Multivariate logistic regression was applied to investigate the independent effect of health-related self-efficacy on lifetime suicidal thought and attempt after separate and successive inclusion of other independent variables in the model. The independent effects of health- related self-efficacy on associations with future suicide intent were performed. The subgroup here was those with life-time suicidal ideation. The model was adjusted for age, gender, marital status, history of psychiatric disorders, substance abuse and self-rated physical & mental health. We modelled whether self-efficacy would be independently associated with different assessments of suicidality (suicide attempt, lifetime suicide ideation and future intent). All statistical analyses were performed using the SPSS v21, and statistical significance was defined as a value of *p* < 0.05.

## Results

We obtained available information on suicide behaviours for the analysis of 2110 participants. Among the cohort, 53.2% were women. People who aged 15–19 years represented 20.1% of the sample; nearly half were those aged between 30 and 60 years (52.2%), and 27.7% was aged 60 years and above. About two-third (65.6%) of the participants was married and 28.2% was single. Sample characteristics were reported in Table 1. The mean (standard deviation) score of health-related self-efficacy was 78.8 (13.4). We stratified the self-efficacy sub-groups based on self-efficacy score tertiles. A low tertile self-efficacy score we defined as below 80. The mean score for low self-efficacy group was 63.8 (10.8), this was significantly lower than rest of the sample 86.1 (6.9), *t* = 55.8, *p* < 0.001. Participants with psychological distress and substance abuse of drug or alcohol represented in 15 and 2.9% of the sample, respectively. One-fourth (25.4%) reported poor self-rated physical health and 14.4% had poor self-rated mental health. Suicidality measured as lifetime suicidal ideation and prior attempted suicide were reported to be 12.6 and 2.7%, respectively. Among those with suicide ideation, 9.8% had thoughts of future intention of suicide.

**Table 1:** Characteristics of the sample (*N* = 2110)

	N (%)
Gender	
Female	1122 (53.2)
Male	988 (46.8)
Age	
15–29	424 (20.1)
30–59	1101 (52.2)
60 & above	585 (27.7)
Education^a^	
Elementary school and below	212 (10.0)
Junior high school	267 (12.7)
Senior/vocational high school	694 (32.9)
Technical college	274 (13.0)
College	545 (25.8)
Graduate school	113 (5.4)
Marital status^a^	
Single/Married	1979 (93.8)
Divorced/Widowed/Separated	126 (6.0)

^a^Missing = 5

### Study factors associated with suicidality

Age and gender were not associated with suicidal behaviours in our sample (Table 2). However a higher education level had a protective effect with lower risk of suicidal attempt (OR = 0.4, 95% CI 0.2–0.7). People divorced or separated compared to those married or single had higher risks of suicidal thoughts (OR = 2.2, 95% CI 1.4–3.4) and attempted suicide (OR = 5.2, 95% CI 2.7–9.9). Psychological distress and substance abuse exposed a strong risk with suicidal behaviours. The risks for suicidal behaviours were about four times higher for those affected by psychopathological symptoms and six times for previous substance abuse. Specifically, the risk of suicidal thought was (OR = 4.3, 95% Cl 3.2–5.7), suicidal attempt (OR = 4.6.1, 95% CI 3.5–10.4) and future suicide intent (OR = 10.9, 95% CI 5.1–22.9) for those with psychopathological symptoms. For substance abuse, the risk for suicidal thought was (OR = 6.0, 95% CI 3.5–10.1); attempted suicide (OR = 6.3, 95% CI 2.8–13.9) and future intent of suicide (OR = 6.4, 95% CI 2.4–17.3).

**Table 2:** Sample characteristics and suicidality

	Life time suicide ideation		OR (95% CI); *p* value	Suicide attempt		OR (95% CI); *p* value	Future suicide intent		OR (95% CI); *p* value
	No n (%)	Yes n (%)		No n (%)	Yes n (%)		No n (%)	Yes n (%)	
Gender									
Female	955 (85.1)	167 (14.9)	Ref	1086 (96.8)	36 (3.2)	Ref	1105 (98.5)	17 (1.5)	Ref
Male	889 (90.0)	99 (10.0)	0.6 (0.5–0.8); *p* = 0.001	968 (98.0)	20 (2)	0.6 (0.4–1.1); *p* = 0.10	972 (98.4)	18 (1.8)	1.0 (0.5–2.1); *p* = 0.86
Age									
15–29	382 (90.1)	42 (9.9)	Ref	415 (97.9)	9 (2.1)	Ref	421 (99.3)	3 (0.7)	Ref
30–59	947 (86.0)	154 (14.0)	1.5 (1.0–2.1); *p* = 0.03	1074 (97.5)	27 (2.5)	1.2 (0.5–2.5); *p* = 0.70	1080 (98.1)	21 (1.9)	2.7 (0.8–9.1); *p* = 0.10
60 & above	515 (88.0)	70 (12.0)	1.2 (0.8–1.8); *p* = 0.30	565 (96.6)	20 (3.4)	1.6 (0.7–3.6); *p* = 0.22	576 (98.5)	9 (1.5)	2.1 (0.6–8.1); *p* = 0.24
Education									
School level	1016 (86.6)	157 (13.4)	1.0	1130 (96.3)	43 (3.7)	1.0	1152 (98.2)	21 (1.8)	1.0
College & above	828 (88.4)	109 (11.6)	0.9 (0.7–1.1); *p* = 0.23	924 (98.6)	13 (1.4)	0.4 (0.2–0.7); *p* = 0..001	925 (98.7)	12 (1.3)	0.7 (0.3–1.5); *p* = 0.38
Marital status									
Single/Married	1742 (88.0)	237 (12.0)	1.0	1936 (97.8)	43 (2.2)	1.0	1947 (98.4)	32 (1.6)	1.0
Divorced/Widowed/ Separated	97 (77.0)	29 (23.0)	2.2 (1.4–3.4); p = 0.001	113 (89.7)	13 (10.3)	5.2 (2.7–9.9), *p* < 0.001	125 (99.2)	1 (0.8)	0.5 (0.06–3.5); *p* = 0.71
Substance abuse									
No	1810 (88.3)	239 (11.7)	1.0	2001 (97.7)	48 (2.3)	1.0	2021 (98.4)	28 (1.4)	1.0
Yes	34 (55.7)	27 (44.3)	6.0 (3.5–10.1); p < 0.001	53 (86.9)	8 (13.1)	6.3 (2.8–13.9); *p* < 0.001	56 (91.8)	5 (8.2)	6.4 (2.4–17.3); *p* = 0.002
Psychological distress									
No	1608 (90.6)	166 (9.4)	1.0	1746 (98.4)	28 (1.6)	1.0	1763 (99.4)	11 (0.6)	1.0
Yes	217 (69.1)	96 (30.9)	4.3 (3.2–5.7); p < 0.001	286 (91.1)	28 (8.9)	6.1 (3.5–10.4); *p* < 0.001	132 (89.2)	20 (6.4)	10.9 (5.1–22.9); *p* < 0.001
Poor Self-rated mental health									
No	1616 (89.9)	181 (10.1)	1.0	1767 (98.3)	30 (1.7)	1.0	1784 (99.3)	13 (0.7)	1.0
Yes	221 (72.7)	83 (27.3)	3.3 (2.5–4.5); p < 0.001	279 (91.8)	25 (8.2)	5.3 (3.1–9.1); *p* < 0.001	284 (93.4)	20 (6.6)	9.7 (4.7–19.6); *p* < 0.001
Poor self-rated physical health									
No	1421 (90.7)	145 (9.3)	1.0	1538 (98.2)	28 (1.8)	1.0	1552 (99.1)	14 (0.8)	1.0
Yes	418 (78.0)	118 (22.0)	2.7 (2.1–3.6); p < 0.001	509 (95.0)	27 (5.0)	2.9 (1.7–4.9); *p* < 0.001	518 (96.6)	18 (3.4)	3.9 (1.9–7.9); *p* < 0.001
Health self-efficacy score									
> =80	1265 (91.9)	111 (8.1)	Ref	1358 (98.7)	18 (1.3)	Ref	1371 (99.6)	5 (0.4)	Ref
< 80	515 (77.9)	146 (22.1)	3.2 (2.5–4.2); p < 0.001	627 (94.9)	34 (5.1)	4.1 (2.2–7.3)	635 (96.1)	26 (3.9)	11.2 (4.2–29.3)

*OR (95% CI)* Odds Ratio (95% Confidence Interval)

Poor self-rated mental health and physical health also showed associations with suicidality. The risk for suicidal ideation among those with poor self-rated mental health was (OR = 3.3, 95%CI 2.5–4.5). The risk increases for attempted suicide (OR = 5.3, 95% CI 3.1–9.1) and future suicide intent (OR = 9.7, 95% CI 4.7–19.6). For those with poor self-rated physical health the risk for suicidal ideation was (OR = 2.7, 95% CI 2.1–3.6), suicidal attempt (OR = 2.9, 95% CI 1.7–4.9) and future intent of suicide (OR = 3.9, 95% CI 1.9–7.9).

### Health-related self-efficacy and suicidality

Low health self-efficacy was associated with suicidal behaviours. The risks for suicidal ideation, attempted suicide and future suicide intent were (OR = 3.2, 95% CI 2.5–4.2); (OR = 4.1, 95% CI 2.3–7.3) and (OR = 11.2, 95% CI 4.2–29.3) respectively. Tables 3 & 4 explained the stepwise logistic regression and factor adjustments. These factor adjustments were conducted individually and sequentially to investigate the independent association between low health-related self-efficacy and suicidal behaviours. Low health-related self-efficacy was associated with lifetime suicide ideation after controlling for gender, age, education, marital status, substance abuse, psychological distress, poor mental and physical health (OR = 2.0, 95% CI 1.5–2.8). Low health self-efficacy was associated with suicidal attempt after adjustment. The association weakened when sequential analysis of the factors self-rated mental and physical health were added to the models (OR = 1.9, 95% CI 1.0–3.7).

**Table 3:** Logistic regression for the association between low health self-efficacy and lifetime suicide ideation

	Life-time suicide ideation			
	Individual adjustment^a^		Sequential adjustment^b^	
	OR (95% CI)	χ2 (df) p	OR (95% CI)	χ2 (df) p
Unadjusted	3.2 (2.5–4.2)			
Gender	3.3 (2.5–4.3)	75.8 (1) < 0.001	3.3 (2.5–4.3)	75.8 (1) < 0.001
Age	3.3 (2.5–4.3)	74.4 (1) < 0.001	3.3 (2.5–4.3)	74.4 (1) < 0.001
Education	3.3 (2.5–4.3)	73.9 (1) < 0.001	3.3 (2.5–4.3)	73.9 (1) < 0.001
Marital status	3.1 (2.4–4.1)	70.0 (1) < 0.001	3.2 (2.5–4.3)	71.8 (1) < 0.001
Substance abuse	2.9 (2.3–3.9)	61.6 (1) < 0.001	2.9 (2.3–3.9)	60.4 (1) < 0.001
Psychological distress	2.6 (2.0–3.5)	47.5 (1) < 0.001	2.5 (1.9–3.3)	38.8 (1) < 0.001
Poor self-rated mental health	2.6 (1.9–3.4)	42.6 (1) < 0.001	2.2 (1.6–3.0)	26.9 (1) < 0.001
Poor self-rated physical health	2.5 (1.8–3.3)	39.4 (1) < 0.001	2.0 (1.5–2.8)	20.4 (1) < 0.001

*OR (95% CI)* Odds Ratio (95% Confidence Interval)

^a^Individual adjustments: adjusted for gender, age, marital status, etc.; ^b^sequential adjustments: adjusted for gender, gender + age, gender +age + marital status, etc

**Table 4:** Logistic regression for the association between low health self-efficacy and suicide attempt

	Attempted suicide in the past			
	Individual adjustment^a^		Sequential adjustment^b^	
	OR (95% CI)	χ2 (df) p	OR (95% CI)	χ2 (df) p
Unadjusted	4.1 (2.3–7.3)	22.7 (1) < 0.001		
Gender	4.1 (2.3–7.4)	23.1 (1) < 0.001	4.1 (2.3–7.4)	23.1 (1) < 0.001
Age	3.9 (2.2–7.1)	21.5 (1) < 0.001	4.0 (2.2–7.2)	21.9 (1) < 0.001
Education	3.8 (2.2–6.9)	20.9 (1) < 0.001	3.8 (2.1–6.9)	20.5 (1) < 0.001
Marital status	3.7 (2.0–6.7)	19.7 (1) < 0.001	3.7 (2.1–6.6)	18.9 (1) < 0.001
Substance abuse	3.6 (2.0–6.5)	18.4 (1) < 0.001	3.3 (1.8–6.1)	15.7 (1) < 0.001
Psychological distress	3.0 (1.6–5.5)	12.9 (1) 0.001	2.5 (1.4–4.7)	8.5 (1) 0.004
Poor self-rated mental health	2.6 (1.4–4.8)	8.8 (1) 0.003	2.0 (1.0–3.8)	4.4 (1) 0.04
Poor self-rated physical health	2.9 (1.6–5.6)	11.5 (1) 0.001	1.9 (1.0–3.7)	3.4 (1) 0.07

*OR (95% CI)* Odds Ratio (95% Confidence Interval)

^a^Individual adjustments: adjusted for gender, age, marital status, etc.; ^b^sequential adjustments: adjusted for gender, gender + age, gender +age + marital status, etc

Among those with lifetime suicidal ideation, stepwise logistic regression demonstrated an association between low health self-efficacy and future suicide intent (Table 5). Low health self-efficacy was independently associated with future intent even after adjustment of gender, age, education, marital status, substance abuse, psychological distress, poor mental and physical health (OR = 4.8, 95% CI 1.0–22.6). We repeated the analyses with self-efficacy as a continuous variable and the association between self-efficacy score and suicidality remained unaltered.

**Table 5:** Logistic regression for the association between low health self-efficacy and future suicide intent

	Future suicide intent			
	Individual adjustment^a^		Sequential adjustment^b^	
	OR (95% CI)	χ2 (df) p	OR (95% CI)	χ2 (df) p
Unadjusted	9.6 (2.2–42.0)	9.1 (1) 0.002		
Gender	9.4 (2.1–41.1)	8.9 (1) 0.003	9.4 (2.1–41.1)	8.9 (1) 0.003
Age	9.6 (2.2–41.7)	9.0 (1) 0.003	9.3 (2.1–40.9)	8.8 (1) 0.003
Education	9.5 (2.2–41.7)	9.0 (1) 0.003	9.3 (2.1–40.6)	8.7 (1) 0.003
Marital status	10.0 (2.3–43.7)	9.4 (1) 0.002	9.5 (2.2–41.8)	8.9 (1) 0.003
Substance abuse	9.6 (2.2–41.9)	9.0 (1) 0.003	9.4 (2.1–41.5)	8.8 (1) 0.003
Psychological distress	6.5 (1.5–29.0)	6.0 (1) 0.01	5.9 (1.3–27.2)	5.4 (1) 0.02
Poor self-rated mental health	7.1 (1.6–31.9)	6.7 (1) 0.01	5.6 (1.2–25.7)	5.0 (1) 0.03
Poor self-rated physical health	7.3 (1.6–33.0)	6.8 (1) 0.009	4.8 (1.0–22.6)	4.9 (1) 0.04

*OR (95% CI)* Odds Ratio (95% Confidence Interval)

^a^Individual adjustments: adjusted for gender, age, marital status, etc.; ^b^sequential adjustments: adjusted for gender, gender + age, gender +age + marital status, etc

## Discussion

In a large community-based telephone interview in Taiwan, we demonstrated that lower levels of health-related self-efficacy were associated with suicidality (i.e., lifetime suicidal ideation, past suicidal attempts and having future intention of suicide). The study findings were generally typical of the literature globally and for the region. In our sample, 12.6% had suicidal ideation and 2.7% attempted suicide. Globally, the estimated lifetime prevalence (SE) of suicidal ideation, plan, and attempt in cross-national sample is 9.2% (0.1), 3.1% (0.1), and 2.7% (0.1) [19]. In the year 2010, a weighted prevalence of 18.5% was reported for lifetime suicidal ideation in a nationwide community survey conducted using a computer-aided telephone interview system with residents aged ≥15 years in Taiwan [2].

Our study and others have shown that female gender, low education, people living alone or separated, history of psychiatric disorders and substance abuse were associated with suicidality [20–24]. In particular we found female gender was associated with lifetime suicidal thought but not attempt and this may reflect higher prevalence of depression among women than men [25]. While this may seem paradoxical, women in Asian countries are more likely to seek treatment help for depression compared to male counter parts [26]. Low-education may result in socio-economic disadvantage, unemployment and low-income and in turn increase suicidal risk [4, 27]. Moreover, living alone is a predictor of suicidality, and it is proposed that the diminished family connectedness interacts with suicidal behaviours [28]. In our study and others, it is summarized that the presence of psychiatric disorders and substance abuse convey the highest risk for suicide [20–24].

We demonstrated an increased risk of lifetime suicidal thought or suicidal attempt with low self-efficacy was independent of perceived mental and physical health. Importantly, among those with lifetime suicidal thought, health-related self-efficacy was an independent predictor of having thoughts for suicide actions in the future. A growing body of evidence supports the relationship between self-efficacy and sense of personal control over behaviour change. Indeed self-efficacy has an important role in patient health outcomes [29, 30]. Self-efficacy has been identified as a significant factor explaining the benefits of treatment and health promoting behavioural change in smoking cessation, alcohol and weight loss and chronic disease self-management [31–33]. Health self-efficacy reflects individual’s coping ability and confidence in their ability to take care of their health in different circumstances [16]. As health self-efficacy indicates the perceived ability to challenge and cope with adverse situations and develop a sense of health control, it is possible for health self-efficacy to have a direct or indirect effect (difficulty coping with stress and health problems) on suicidal behaviour [34].

The sensitivity of the association between health self-efficacy and suicidality was confirmed in our subgroup analyses. For example, among those with lifetime suicidal ideation, low health self-efficacy was associated with future suicide intent. Hence, health self-efficacy assessment may be a sensitive cognitive substrate to screen people with predisposed risk factors for suicide. Moreover, health self-efficacy assessment is simple and can be used by health professionals without much stigmatisation associated with binary mental health assessment scales. Increasing self-regulation, planning behaviour, positive feedback and empowerment can increase self-efficacy in carrying out lifestyle changes. There is evidence that mental health self-efficacy influences symptom outcomes when clients use a self-guided mobile phone and web-based psychotherapeutic intervention [35]. However longitudinal studies are needed to confirm the causal associations between the changes in health self-efficacy and suicidal behaviour in the longer term. Future research could also assess the potential of health self-efficacy interventions in reducing suicidal thoughts and further risks of suicide, such as future intent or prior attempts of suicide.

Our study has salient strengths. The CATI survey has been conducted by the TSPC over a decade. The findings were derived from a large sample size randomly selected, thus ensuring representativeness. In this study, we simultaneously measured suicidality outcomes (lifetime/recent suicidal thought, prior attempt and future intent) in a random sample. Given that the topic is sensitive and complex, the responders could reply to these questions more comfortably via telephone interview due to anonymity. We controlled from major confounding factors known to be associated with suicidality, including self-perception of mental health and physical health. This emphasizes that health self-efficacy is an independent construct that is not superimposed or biased by self-perception of health. The main limitation of the study was that the findings were based on cross-sectional analyses, hence the causal inference could not be achieved. It is possible that participants with lower health-related self-efficacy may have difficulties managing their health and indulge in risky behaviours such as substance abuse. Indeed substance abuse is associated with suicidal behaviours. We had not measured factors such as whether lower health literacy was associated with reduced health-related self-efficacy and suicidal behaviours. On the other hand as we report in our study lower self-efficacy may serve as a marker for suicidal behaviours. Further, telephone interviews have innate limitations. For example, we drew landline numbers based on the registration list in computer directories and did not include mobile phone users, thus the results could only generalize to people who have landlines. However, CATI is a professional technique in collecting research data. Although such interviews may be influenced by participant environmental factors, which the researcher has limited control, structured questions and standard operating procedures developed by the researchers at the TSPC were followed by experienced personnel with specific training, thus ensuring that reliable data were acquired. Moreover, the research team has careful inspection of the yearly results drew from CATI for more than 12 years and published articles elsewhere [2, 18], which provide evidence of the reliability of our study.

Regardless of these limitations, the study has implications for identifying and intervening individuals with suicidal risk. We have shown for the first time an association between health-related self-efficacy and lifetime suicidal thought and behaviour in Taiwan; and that the association was robust and was independent of factors known to be associated with suicidality. The authors concluded that health-related self-efficacy was associated with suicide risks in different time points. Perceived efficacy in health was significantly affected current or prior suicide ideation and future suicide intent. Our study adds to the literature on potentially modifiable factors and that evaluation of the progress of self-efficacy in health may be long-term targets of intervention in suicide prevention strategies.

## Abbreviations

CATI: Computer-assisted telephone interview
CIs: Confidence intervals
ORs: Odds ratios
TSPC: Taiwan Suicide Prevention Centre
